# Comparison of three full-field optical measurement techniques applied to vibration analysis

**DOI:** 10.1038/s41598-023-30053-9

**Published:** 2023-02-24

**Authors:** Patrick O’Donoughue, François Gautier, Erwan Meteyer, Thomas Durand-Texte, Mathieu Secail-Geraud, Félix Foucart, Olivier Robin, Alain Berry, Manuel Melon, Charles Pézerat, Adrien Pelat, Pascal Picart

**Affiliations:** 1 Centre de Recherche Acoustique-Signal-Humain, Université de Sherbrooke, 2500 Boulevard de l’Université, Sherbrooke, QC Canada; 2 Laboratoire d’Acoustique de l’Université du Mans (LAUM), UMR 6613, Institut d’Acoustique-Graduate School (IA-GS), CNRS, Le Mans Université, Avenue Olivier Messiaen, 72085 Le Mans France

**Keywords:** Acoustics, Imaging techniques, Optical techniques, Imaging techniques, Mechanical engineering

## Abstract

Digital image correlation, deflectometry and digital holography are some of the full-field optical measurement techniques that have matured in recent years. Their use in vibroacoustic applications is gaining attention and there is a need for cataloging their performance in order to provide, to a broad community of users and potential future users, quantitative and qualitative evaluations of these three approaches. This paper presents an experimental comparison of the three optical methods in the context of vibration measurements, along with classical reference measurements provided by an accelerometer and a laser Doppler vibrometer. The study is carried out on two mechanical structures exhibiting various vibration responses when submitted to an impact.

## Introduction

In many domains, such as ground, naval or air transportation, structural vibrations are closely related to mechanical reliability and noise sources. Vibrations can be generated by different excitation types: mechanical, acoustical, aerodynamic, magnetic, etc. The understanding of vibratory phenomena is generally carried out via the analysis of operational vibration responses, which correspond to the vibratory field resulting from the excitations in the practical context. The operational vibration responses are useful to determine vibratory transfer paths, perform a modal analysis of the structure, identify excitation sources or predict radiated noise. Therefore, the vibration field is the basic input data for such applications. In vibration and structural acoustic applications, the vibration wavelengths of interest are generally in the centimeter to meter range. A full-field vibration measurement of a surface will typically require 6 to 10 points per wavelength to ensure adequate spatial sampling and therefore may imply a very large number of data points on large structures. From an experimental point of view, several more or less sophisticated approaches may provide vibration fields.

The accelerometer is certainly the most utilized sensor for vibration measurements in the academic and industrial worlds due to its robustness, sensitivity, wide bandwidth and high dynamic range. However, it only yields point-wise measurements of the vibratory field at the location where it is attached to the structure. In order to obtain a collection of vibration data points, it is therefore necessary to repeat the measurement by moving the sensor, or to increase the number of sensors while increasing the overall complexity of the setup. In addition, the behavior of the structure can be locally modified by the added mass of the accelerometer, and added damping from the attached cable. In the majority of applications, the accelerometer mass is chosen such that the structure suffers from a small enough perturbation. Nevertheless, accelerometers are always intrusive and even more so in the case of lightweight structures.

The significant progress in laser technology and instrumentation have led to the development of contact-less measurements with the laser Doppler vibrometry (LDV). The basic principle is the Doppler frequency shift of the reflected laser frequency due to the motion of the measured surface. The laser Doppler vibrometer therefore provides velocity measurements along the beam direction^1–4^. The main interest of the laser vibrometer is for measuring the vibratory field without any contact and without any intrusion at the surface of the structure. In addition, the development of scanning laser Doppler vibrometer adds the possibility of obtaining a collection of data points at the inspected surface^5–8^. In this respect, previous works reported the scanning of 256 points along one line up to 80 kHz^5,6^, the use of holographic optical elements associated with one CMOS sensor (vibration measured up to 100 kHz^7^), the use of frequency multiplexing (20 points with $5 \times 4$ beams^8^), or the use of three acousto-optical devices and a single high-speed photodetector ($5 \times 4$ beams with a rate of 500 Msamples/s^9^). Although these approaches yield a set of independent measurements at several points on the surface, the number of simultaneous measurements remains limited. In order to acquire the vibration response at many points, laser Doppler vibrometry requires a repetition of the measurement, and therefore, the use of controlled and repeatable excitation sources. Various evolutions emerged in recent years, such as the 3D extension of scanning laser Doppler vibrometer in order to measure all 3 components of the vibration field, with possible coupling with a robotic arm^10^. Such tools are very powerful for vibration analysis of complex structures, but they are costly.

The simultaneous collection of a large number of data points at the surface of dynamic structures can be obtained by other existing approaches based on optical imaging. As a result of this full-field data recording, the acquisition time is independent of the number of measurement points, enabling dense spatial measurements to be performed in a fraction of the time required for a classical scanning vibrometer.

Stereo digital image correlation^11–13^ yields measurements over large structures for movements or deformations with high amplitude. It is non-intrusive, full-field, and applicable to a wide range of geometries. In the context of vibration measurements, 3D vision methods with high-speed cameras based on the concepts of dynamic photogrammetry have been adapted to measure structural vibrations^14–16^. As high-speed cameras are expensive and require accurate synchronization, the unconventional single-camera pseudo-stereo system was proposed, for which the camera sensor is split into two halves, thus generating two virtual cameras. The digital image correlation (DIC) method coupled to this pseudo-stereo setup and a single high-speed camera has recently been used to measure the vibrations of a plate in comparison to a reference technique^15^. Since this method is based on intensity changes in images, the sensitivity is less than laser Doppler vibrometry. On the other hand, low-frequency and high amplitude displacements can be targeted. For vibration measurements, digital image correlation coupled with a single high-speed camera and dedicated triangulation methods has provided insightful results for transient signals^16^.

In the late 1900s, parallel development of the deflectometry technique occurred in both 3D shape metrology^17^ and solid mechanics^18^. The latter led to applications of deflectometry in material identification^19,20^ and damage detection^21,22^ from full-field-measurements. Deflectometry directly provides a measurement of surface slopes. Deflections and curvatures, that are often needed for calculations in bending wave vibrations, can be obtained by a single spatial integration and differentiation of the measured slope fields, respectively. With the use of high-speed cameras, it follows that both stationary and transient excitation cases can be analyzed^23–25^. In^26^, full-field deflectometry measurements on a metal panel were used to identify stationary point loads and impact forces. Similarly, acoustic and aerodynamic pressure distributions on flat plates were reconstructed from deflectometry data^27,28^. Note that the use of deflectometry requires a specimen surface that is flat and specularly reflective, which corresponds to a mirror-like surface in the visible spectrum. Recent works^29,30^ show that the use of infrared light sources and high-speed infrared cameras may overcome such a limitation.

Full-field evaluation of surface deformation, shape and vibration can also be obtained with coherent imaging. It requires expanded coherent laser beams to produce interferences by mixing with a controlled laser beam (the so-called reference beam). The coherent imaging approach yields a high density of data points and includes a variety of techniques such as shearography, speckle interferometry and digital holography^31,32^. Quantitative methods for vibration retrieval were developed with stroboscopic illumination^33–37^ and the laser-pulse regime^38–43^. As examples, these approaches were applied to vibration of micro-membranes^37^, modal analysis^40,41^, determination of structural intensity^41^, the observation of acoustic waves^40–42^, the high amplitudes of self-oscillations of a clarinet reed^35^, and also shocks^43^. Although being able to provide quantitative data, the recording process requires complex operations such as phase-shifting and laser pulse triggering. More recently, the use of high-speed sensors enabled acquiring holographic data of the time-evolution of the studied phenomena^42,44–50^. The advantage is that the optical assembly is considerably simplified, as it does not require a pulsed laser, double pulse laser, or any generation of strobe light pulses.

These three full-field optical measurement techniques, DIC, deflectometry and digital holography, are becoming increasingly popular in academic research and will likely strongly impact the industry in the near future, even if, for now, LDV remains the key tool for contact-less vibration measurements. Nevertheless, as the three methods gain more and more maturity, there is a growing need for cataloging them in terms of performance and ease of setup. Therefore, this paper aims at providing, to a broad community of users and potential future users, qualitative and quantitative evaluations of the three methods with comparison to the classical accelerometer and laser Doppler vibrometer.

## Basic principles of the measurement techniques

### Laser Doppler vibrometry

**Figure 1:**
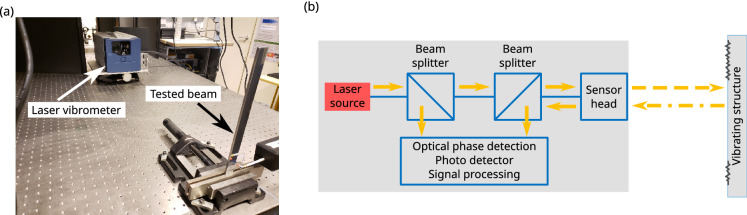
(**a**) Photograph of the laser vibrometry setup used for the measurements, (**b**) diagram of the typical laser vibrometry principle and processing chain.

Laser Doppler vibrometry (LDV) is a widespread technique that enables non-contact measurement of vibrations^1–8^, and finds its root in fluid velocity measurements that were performed in as early as the 1960s^51^. Most of the commercially available systems use a single beam from a low-power laser source, and the technique is based on coherent detection of the Doppler frequency shift that occurs when the laser light is scattered by a vibrating surface. Figure 1a presents a picture of the laser vibrometer measurement setup on an optical table, with the laser vibrometer and the studied structure in the foreground. The usual laser vibrometry principle is reminded in Fig. 1b. Surface vibration velocity at each measurement point is directly obtained, and can further be integrated or differentiated as a function of the frequency to compute displacement or acceleration, respectively. Vibration maps can be obtained using several spatially distributed measurements points. The Doppler frequency shift is directly proportional to the surface velocity, and allows a non-contact measurement of the vibration velocity. Two review papers of this technique can be found in^51,52^.

Apart from very specific issues linked to sensitivity to external or environmental vibrations^53^ that is encountered in several measurement techniques, this method has proved to be reliable and to provide high sensitivity with a large dynamic range. Its classical point-scanning operation is convenient to study stationary or phase-referenced excitations, but many real-world cases and procedures such as impact testing involve non-stationary vibration fields. LDV has thus notably evolved since its invention to tackle these specific issues^54^, from multi-point approaches^55^ to continuous scanning LDV^56^. Introduced in the 1990s, the continuous scanning technique modified the traditional paradigm of LDV, in which the number of measurement points defines the spatial resolution of the measurement map. A stepped-scan approach is now replaced by a continuous-scan approach (a set of fixed-point measurements is replaced by a continuously sweeping trajectory covering the same surface area). Finally, apart from 3D-LDV that usually relies on the combination of three LDV heads, the use of a robot arm with a single LDV has recently proved to be an efficient approach for performing 3D full-field measurements^57^. In this work, LDV, along with an accelerometer, serves as a reference method to which the three full-field optical measurement methods will be compared.

### Digital holography

Digital holography is a general method for imaging and metrology^32,58–60^ and is used for many applications such as microscopy, 3D tomography, surface topography and roughness or surface deformation measurement. By using a high-speed camera sensor for dynamic measurements, digital holography can provide information related to the instantaneous vibration displacement of any structure^36–39,49^. Recent applications^50,61^ have shown that holographic vibration measurements can achieve high spatial and temporal resolutions.

**Figure 2:**
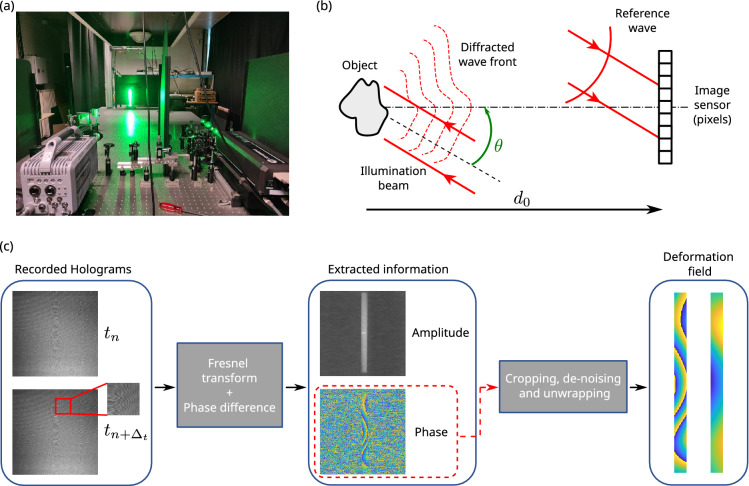
(**a**) Photograph of the holographic assembly, (**b**) basic scheme for digital Fresnel holography; the wave diffracted from the object propagates in the free space to the sensor area, and the reference wave impacts directly the matrix of pixels, (**c**) diagram of holographic image processing.

Figure 2a presents a photograph of the holographic measurement setup on its optical table, with the high-speed camera, optical components and the structure of interest in the background. Digital holography is based on the coherent mixing of two waves. The first wave is a reference laser beam and the second wave is from diffraction of the laser beam by the targeted structure, refer to Fig. 2b. Figure 2c shows a diagram of holographic image processing. The global illumination at the sensor plane is expressed as:

1 $$\begin{aligned} \mathscr {H} = |\mathscr {R}|^2 + |\mathscr {O}|^2 + \mathscr {R}^*\mathscr {O}+ \mathscr {R}\mathscr {O}^*. \end{aligned}$$

In Eq. (1), $\mathscr {H}$ is the recorded hologram resulting from the interference of the reference wave $\mathscr {R}$ and the object wave $\mathscr {O}$. The reconstructed image $\mathscr {I}$ is calculated with the discrete Fresnel transform^62^ defined in Eq. (2) (FFT means Fast Fourier Transform).

2 $$\begin{aligned} \mathscr {I}=h_{F} \times \text {FFT}\left[ \frac{\mathscr {H} - |\mathscr {R}|^2}{\mathscr {R}^*} \times h_{F}\right] , \end{aligned}$$

with $h_F$ the Fresnel kernel defined in the object plane (*x*, *y*) given by Eq. (3).

3 $$\begin{aligned} h_{F}(x, y)=\frac{1}{\sqrt{\lambda _0 d_{r}}} \exp \left( i \pi \frac{d_{r}}{\lambda _0}-i \frac{\pi }{4}\right) \exp \left[ \frac{i \pi }{\lambda _0 d_{r}}\left( x^{2}+y^{2}\right) \right] . \end{aligned}$$

In Eq. (3), $\lambda _0$ is the wavelength of the laser, $d_r$ is the reconstruction distance in the Fresnel transform and $d_0$ is the distance between the measured structure and the image sensor. As a general rule, the image of the object is obtained for $d_r=-d_0$.

The Doppler phase $\Delta \psi _{n}(x,y)$ related to the difference of displacement is then extracted by subtracting the phases of the complex images $\mathscr {I}$. In the case of vibration measurements, this phase difference occurs between consecutive instants at a high frame rate. In short, the phase difference is proportional to the displacement of the object between the two instants. However, the extracted phase difference can be advantageously converted into the instantaneous velocity $V_h^n(x,y)$, knowing the frame rate of the camera $f_e$ as expressed in Eq. (4).

4 $$\begin{aligned} V_h^n(x,y) \approx \frac{\lambda _0 f_e}{2 \pi (1+\cos \theta )} \Delta \psi _{n}(x,y). \end{aligned}$$

In Eq. (4), $\theta$ is the illumination angle as in Fig. 2b. In the case of small displacements (nm to $\mu$m range) the subtraction can be performed with a fixed reference phase and the absolute vibration displacement is obtained. In this study, for added robustness to larger displacements, the instantaneous velocity is considered (refer to Eq. (4)).

Since holographic imaging allows the object area to be reconstructed, several post-processing steps must be applied to the extracted data as depicted in Fig. 2c. First, the useful part is cropped in the reconstructed area. Secondly, de-noising needs to be carried out in order to remove the speckle decorrelation noise. The two-dimensional windowed Fourier transform (WFT2F) algorithm^63,64^ consisting in applying a threshold in the Fourier domain (considered as one of the most efficient filters for fringe pattern analysis^65^) is used to extract the de-noised wrapped phase map at each instant. Finally, phase unwrapping is applied when the displacement of the structure between the two instants is larger than almost half the wavelength of the laser source. The unwrapping algorithm used in this paper is based on least squares minimization^66,67^.

### Uni-axial Digital Image Correlation (U-DIC)

Over the past few decades, methods using Digital Image Correlation (DIC) tools have soared along with the development of industrial digital cameras^68^. Initially, DIC was mainly used to measure in-plane deformations. However, DIC has also been applied, in recent years, to measure out-of-plane displacements induced by vibrations^11,13^.

**Figure 3:**
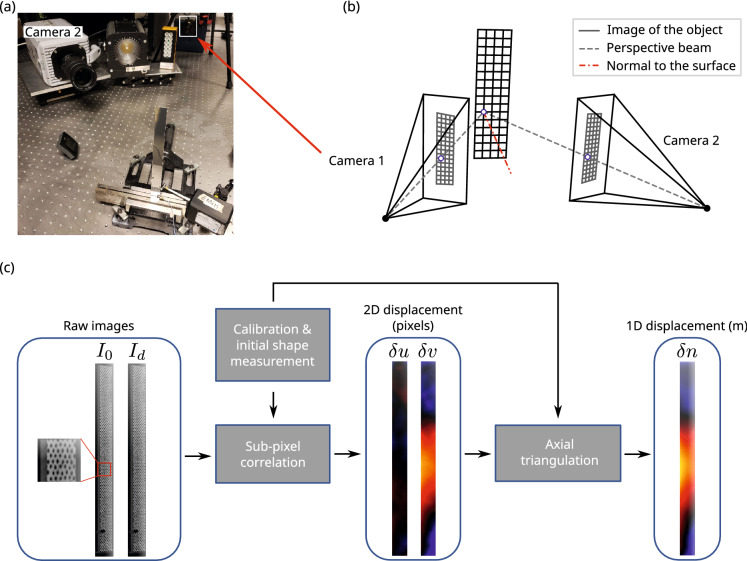
(**a**) Image of U-DIC setup, (**b**) sketch of the triangulation principle, (**c**) diagram of U-DIC processing chain.

When measuring positions and displacements in 3D space, the calculation is based on the triangulation principle: once the camera’s relative position and orientation are determined with calibration procedures, a 3D location is obtained from 2D positions in each image coordinate system (Fig. 3b). The 2D locations, (*u*, *v*), in images are obtained with image processing, namely DIC tools^69^. For an image sequence, the initial (*u*, *v*) are chosen in a reference image $I_0$ and local displacements in images ($\delta u, \delta v)$ are calculated on each deformed image $I_d$ (see Fig. 3c). To do so, a random pattern has to be projected or painted onto the target surface. The displacements are measured on the surface using polynomial interpolation functions. The spatial resolution is thus linked to the random pattern, the size of the surface in the images and the interpolation functions.

The technique requires two viewpoints. If two high-speed cameras are available, 3D vibration measurements are obtained. If a single high-speed camera is used, several measurement methods are available to split the image into two views: using 2- or 4-mirror adapters^15,70,71^, or biprisms for example. However, splitting the image into two views reduces the number of pixels available per viewpoint and hence, lowers the precision of the measurement.

For bending wave vibration measurement, the vibrations occur principally along a single axis: the local normal to the surface. Therefore, a single high-speed camera can be used. For each measurement point, the first triangulation line is the perspective beam obtained via 2D position in the image, and the second one is the local normal estimated from an initial shape measurement (see Fig. 3b)^16^. The obtained measurand is thus the displacement normal to the surface, $\delta n$. This method, here named Uni-axial DIC (U-DIC), is the one employed in this study. The high-speed camera (Camera 2 in Fig. 3a) is used both for shape and vibration measurement, whereas a second low frame rate camera is only used for shape measurement (Camera 1 in Fig. 3a).

Note that the sensitivity for out-of-plane displacement is linked to the angle between the displacement axis and the optical axis of the camera^69,72^, the number of pixels available and the quality of the speckle pattern, but is also related to the size of the measured surface.

### Deflectometry

Deflectometry is a full-field slope measurement technique based on recording the specular reflection of a reference grid on the surface of a plane test specimen. It has been applied to various mirror-like surfaces requiring different levels of preparation. Some materials such as acrylic^19,23^ and glass^25,28^ can be imaged directly without preparation. In^24,26,27^, a polished metal panel was used. Recent works also demonstrated the principle of infrared deflectometry to directly study unpolished metallic plates^29,30^. For other materials, coatings like epoxy resin^73^ and reflective adhesive films^74^ can be considered.

**Figure 4:**
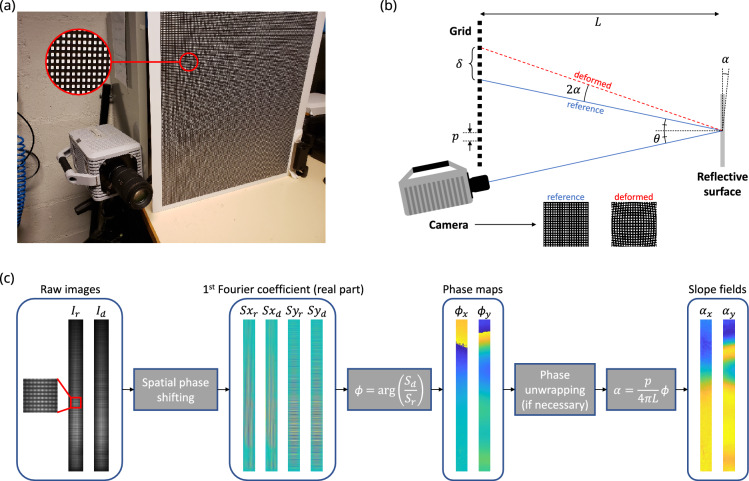
(**a**) Photograph of the deflectometry setup, (**b**) illustration of deflectometry principle, (**c**) diagram of deflectometry processing.

The experimental setup used in this study is depicted in Fig. 4a. The grid is printed with a known line-spacing *p* and placed at a distance *L* from a specularly reflective target structure.

Any flexural surface vibrations will distort the reflected grid image recorded by the camera (see Fig. 4b). The corresponding spatial distortions can be directly related to the local slopes $\alpha _{x,y}$ of the specimen using geometrical considerations (*i.e.*, $\alpha _x(x,y) = \frac{\partial w}{\partial x}(x,y)$, where *w*(*x*, *y*) is the out-of-plane displacement, and similarly for $\alpha _y$). In the small angle approximation for $\alpha$ and $\theta$ (the viewpoint angle relative to the structure normal), the optical intensity variation between the reference and deformed states recorded at a given pixel is attributed to a local shift in the observed position on the grid over a distance $\delta = 2L\alpha$ (refer to Fig. 4b). This distance is further related to the local spatial phase variation $\phi$ identified at the corresponding pixel in the grid images as $\delta = \frac{p}{2\pi }\phi$. The phase variations are obtained by employing a spatial phase shifting approach. A windowed discrete Fourier transform is performed via a 2D convolution^26,27,29,75^. The convolution kernel is detailed in the cited works and has a size of $(2N-1)$ pixels, where *N* is the number of pixels per grid period in the recorded image. A tuning requirement of this phase extraction approach is that *N* must be approximately an integer, which is achieved by physically adjusting the experimental setup.

By combining the two formulas for $\delta$, the resulting phase maps $\phi _{x,y}$ are directly related to the slope fields on the surface of the structure $\alpha _{x,y}$ using the following geometrical relation:

5 $$\begin{aligned} \alpha _{x,y}=\frac{p}{4\pi L}\phi _{x,y}. \end{aligned}$$

The deflectometry image-processing chain is illustrated in Fig. 4c. Calculating out-of-plane displacement maps requires an additional spatial integration operation of the measured slope fields, carried out here using sparse approximation. The point considered for defining the integration constant is chosen among the measurement points that have a supposed null displacement (clamped or simply supported boundary). Alternatively, the displacement at one arbitrary point in the observed region can be measured using a secondary instrument such as a single-point laser Doppler vibrometer.

### Conventions and notations

Table 1 lists the quantities measured with the 5 techniques considered in the study. Each of them is associated with a color code used throughout this paper. The greyed boxes define the basic measured quantities (measurands) and the subscripts refer to the methods, noted *a*, *v*, *h*, *u*, *d*, respectively, for the accelerometer (*a*) with measured acceleration $A_a$, the vibrometer (*v*) with measured velocity $V_v$, holography (*h*) with measured velocity $V_h$, uni-axial DIC (*u*) with measured displacement $X_u$, and deflectometry (*d*) with measured slopes $Sx_d$ and $Sy_d$ according to the *x* and *y* directions. For each method, the transition from one physical quantity to another is performed by means of the indicated relations, which will be used in the following to enable comparison of the measured out-of-plane displacement.

**Table 1:**
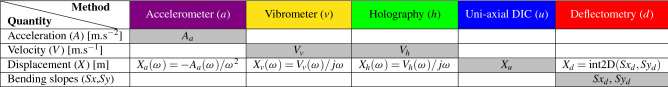
Notations for physical quantities and measurement methods. The methods are color-coded and the measurand of each method is highlighted. Note that int2D denotes the 2D spatial integration used to calculate the displacement field from the measured deflectometry slope fields.

## Experimental methods

### Studied structures and test conditions

**Figure 5:**
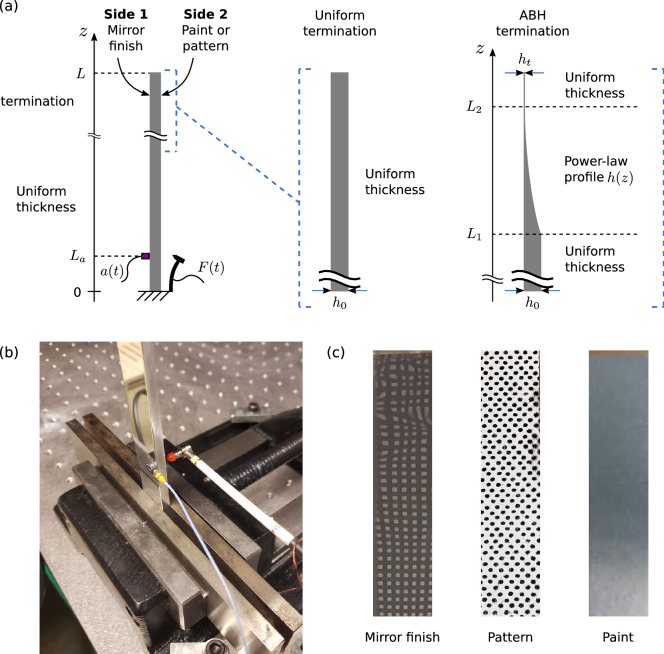
(**a**) Illustration of the uniform and non-uniform mechanical beam used as test structures, (**b**) close-up view of the accelerometer and impact hammer, (**c**) photograph of the termination of the non-uniform beam for the considered three surface conditions; values of the parameters in Fig. 5**a** for both beams are given in Table 2.

This study focuses on the measurement of out-of-plane vibration of cantilever beams. Two structures are used to compare the measurement techniques. The first one in Fig. 5a is the uniform termination configuration and is an aluminum beam of uniform cross-section (dimensions given in Table 2). The second (Fig. 5a) is the Acoustic Black Hole (ABH) termination configuration and is an aluminum beam of variable thickness whose profile is given by Eq. (6).

6 $$\begin{aligned} h(z)=\left\{ \begin{array}{lll} h_0 &{} , \text{ for } z \in \left[ 0 ; L_1\right] \\ h_0+ \left( h_t - h_0 \right) \left( \frac{z-L_1}{L_2-L_1}\right) ^2 &{} , \text{ for } z \in \left[ L_1 ; L_2\right] \\ h_t &{} , \text{ for } z \in \left[ L_2 ; L\right] \end{array}\right. \end{aligned}$$

These thickness variations constitute an acoustic black hole, known to be a penetrable, resonant and absorbing scatterer^76^. The thin end of the beam produces large and localized vibration amplitudes as well as short bending wavelengths, which tests the limits of the measurement techniques.

The two beams are clamped at the base and free at the other end. This mechanical setup is kept unchanged and therefore identical for the five types of implemented measurements. Table 2 gives the geometrical properties of the two beams. The advantage of using two such mechanical beams is to demonstrate the advantages and limitations of each technique on both an academic case (uniform beam), as well as a highly contrasted structure (non-uniform beam). The latter case presents a measurement challenge in terms of dynamic range, bandwidth and spatial resolution.

Excitation is achieved by an impact hammer close to the clamped end and the hammer’s handle is flexible to avoid double impacts (see Fig. 5b). The hammer is placed off-center to excite both bending and torsional modes of the beam. Impacts of different amplitudes are used in order to provide an acceptable signal-to-noise ratio for the different measurement techniques.

The sides of the two beams are respectively polished (side 1) and unpolished (side 2), so as to implement either deflectometry on side 1 or one of the other methods on side 2 with various surface preparations. Holography requires a non-depolarizing silver paint, while DIC uses a random painted pattern. Vibrometry is more versatile, meaning that it can be applied directly to the bare metal, or on the prepared surface. A photograph of the various surface conditions is shown in Fig. 5c. The paints are assumed to have negligible effects on the beams’ dynamics.

**Table 2:** Geometrical parameters of the beams corresponding to the variables in Fig. 5a.

Parameter	Structure	
	Uniform beam	Non-uniform (ABH) beam
Total length [mm]	300	266
Clamped length [mm]	36	32
Width [mm]	20	20
Free length (*L*) [mm]	264	234
Start position of the decreasing thickness ($L_1$) [mm]	–	132
End position of the decreasing thickness ($L_2$) [mm]	–	218
Accelerometer position ($L_a$) [mm]	30	12
Uniform thickness ($h_0$) [mm]	5	3
Truncature thickness ($h_t$) [mm]	–	0.22

### Excitation and signal acquisition

The impact force provided by the hammer was adapted to the different techniques. A peak force of $\sim 35$ N was achieved for uni-axial DIC (u) and deflectometry (d) measurements by manually pulling back and releasing the hammer. On the other hand, an automatic impact hammer (Maul-Theet vImpact) producing a peak force of $\sim 5$ N was used for vibrometry (v) and holography (h) measurements. The automatic impact hammer is essential for the vibrometric measurements in order to achieve repeatable impacts at each point of the measurement mesh. This lower force level was also suitable for holographic measurements in order to limit the fringe density in the reconstructed phase between two instants. The impact hammers were equipped with a force sensor (PCB 086E80, sensitivity 22.5 mV/N), which yields the force applied to the structure. A miniature accelerometer (PCB 352C23, sensitivity 5 mV/g, mass 0.2 g) was mounted on the opposite side of the impact position. Force and acceleration signals were sampled at 102.4 kHz using a National Instruments USB-4431 analog-to-digital converter.

**Figure 6:**
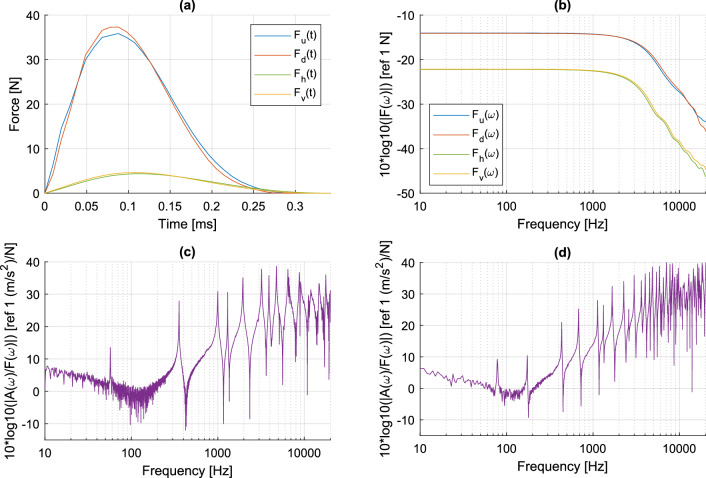
(**a**) Excitation signals; (**b**) power spectrum densities of the excitation signals; (**c**) acceleration/force FRF of the uniform beam from accelerometer measurement; (**d**) acceleration/force FRF of the non-uniform beam from accelerometer measurement.

Figure 6a,b show the time signals and frequency spectra of the impacts, respectively. The frequency response of the impact force is flat up to 1 kHz and its cutoff frequency is slightly higher than 1 kHz. This cutoff frequency is related to the duration of the impact, which depends on the flexibility of the arm and the hardness of the contact surfaces during the impact. Note that a small plastic tip covers the end of the impact hammer; this configuration allows a reasonable compromise between the useful frequency band and the response level. Figure 6c,d show the acceleration/force Frequency Response Function (FRF) derived from the accelerometer for the uniform and non-uniform beam, respectively. Both beams exhibit low-damped vibration modes up to 20 kHz; the black-hole termination of the non-uniform beam induces a higher modal density and larger acceleration in the high frequency range^77^. This shows that the non-uniform configuration is relevant to test the metrological performance of the different techniques.

### Optical measurement configurations

**Figure 7:**
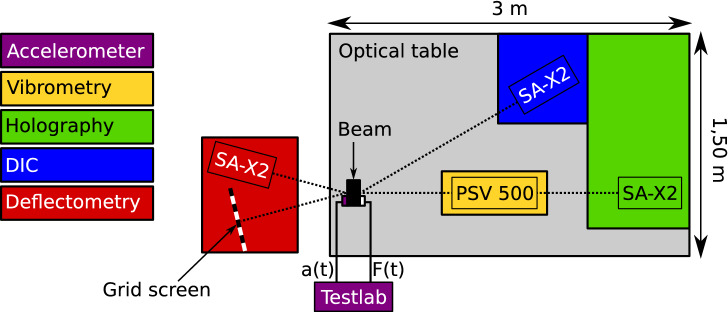
Experimental configuration of the measurement techniques.

All the full-field optical methods used the same high-speed camera (Photron SA-X2 Type 1080K, maximum resolution $1024\,\times \,1024$ pixels up to 12,500 fps, maximum frame rate 1,080,000 fps at the lowest resolution of $128\,\times \,8$ pixels). Depending on the requirements of the methods, the camera was placed at different locations: facing the measured surface for digital holography, with an angle close to 45$^{\circ }$ for uni-axial DIC, and with an angle of few degrees for deflectometry (on the opposite side of the beam, which was polished to a specular finish). A sketch of the positioning of each technique around the tested beam is shown in Fig. 7. Such a configuration is chosen to avoid moving the tested structure between measurements. The measurements were carried out over a period of 2 days for each beam in a temperature controlled room (18 °C).

For the measurements using the high-speed camera, the same software (Photron FASTCAM Viewer) was used to drive the camera and control parameters such as the frame rate, resolution, shutter speed, etc. These parameters were adjusted independently for each technique based on its requirements. The cooling fans of the camera were turned off during acquisition in order to prevent noise and the measurements were synchronised using a Transistor-Transistor Logic (TTL) system that was triggered from the force signal.

All the signals were recorded for a duration of 0.25 s for the uniform beam and 0.5 s for the non-uniform beam. The Fourier analysis is performed using an exponential window, $w(t) = \exp \left( -D t \right)$, with *D* a decay constant arbitrarily fixed at 15 for the uniform beam and at 5 for the non-uniform beam. A force/gate window (short rectangular window isolating the force peak) is applied to the force signal in order to remove artefacts of the hammer dynamics before and after the impact.

#### Laser Doppler vibrometry

A mesh of $185\,\times \,7$ measurements points was used for the uniform and $179\,\times \,21$ points for the non-uniform beam. The measurements were performed using a Polytec PSV 500 Xtra laser Doppler vibrometer with a single impact per measurement point from the automatic hammer (no averaging). A small drift in the performance of the impact hammer was observed; the dispersion of the impacts remains however acceptable. Given the sampling frequency (100 kHz) and the measurement time window, the bandwidth is almost 50 kHz with a frequency resolution of 0.25 Hz.

#### Digital holography

The resolution of the camera was set to 512 $\times$ 512 pixels and the exposure time was 1 μs. The laser power was set to 3.50 W. The frame rate was 40,000 fps both for the uniform and the ABH beam. Referring to Fig. 2b, the distance between the beams and the sensor plane was $d_0 =2.435$ m and a divergent zoom of focal length $-100$ mm was placed at 265 mm from the sensor. These parameters lead to a reconstruction distance $d_0' = 337$ mm. The reconstruction of the beam vibration was performed for a virtual image of dimensions $L_{x}' = L_{y}' \approx 9.1$ mm. The real size of the vibrating surface is then recovered by the use of an optical magnification of $g_{opt} \sim 0.031$. The illumination beam impacts the object surface with angle $\theta = 15$° and the observation was at normal incidence. The number of data points in the reconstructed vibration map depends on the number of points used for the computation of the Fresnel transform. In the case of the uniform beam, the native resolution of the hologram was used for the image reconstruction. In the case of the non-uniform beam, the image reconstruction was performed by doubling the number of data points in the Fresnel transform using zero-padding, which is useful when the vibration amplitude is high and generates many phase jumps^78^.

#### Uni-axial DIC

For the uni-axial DIC method, two cameras were used. The first one is a static camera and was placed in front of the beam, at a distance of 70 cm and at normal incidence. According to the DIC procedure described in^16^, this camera was used for the optical calibration process (compensation for lens distortion) and the estimation of the initial shape of the measured object (stereo-vision approach). The recorded images had a size of $1200 \times 1600$ pixels and the beam was viewed on approximately $90 \times 1100$ pixels. The main Photron high-speed camera was equipped with an adjustable zoom lens (Sigma 17–50 mm f/2.8 EX DC OS HSM) and placed at about 40 cm from the beam with an incidence angle of $45^\circ$ (see Fig. 3a). Image sequences with $104 \times 1024$ pixels were recorded at a frame rate of 40 kHz for the uniform beam and 20 kHz for the non-uniform beam. Note that the choice of the lower frame rate in the latter measurement is due to the response level being below the measurement noise floor above 10 kHz. In order to perform image correlation, random patterns were applied to the two tested beams over a layer of white paint. In order to optimize the accuracy of the measurement, each patch of the pattern has to be imaged over an area of 3 to 8 pixels wide^69^. Two LED spotlights were used to obtain sufficient brightness (about 90% of the saturation intensity of the camera sensor for the brightest pixels). The set of images was recorded by the high-speed camera and then used to calculate the normal displacement of the beams via triangulation.

#### Deflectometry

The grid pattern used in the deflectometry measurements was printed on white signage board with a pitch (line separation) of $p=4$ mm. The grid was placed beside the camera, near the midpoint of the camera lens (Sigma 105 mm f/2.8 EX DG Macro HSM), and illuminated with the two LED spotlights. For the measurement on the uniform beam, the distance of the camera and grid from the beam was adjusted to $L=1.45$ m in order to obtain $N=7$ pixels per grid period in the image. The images were recorded with a resolution of $88 \times 1024$ pixels and at a frame rate of 40 kHz.

For the non-uniform beam, the warping of the metal at the thin ABH termination (visible in the mirror finish of Fig. 5) distorted the initial grid image. The warping occurs after the ABH profile is machined into the beam and is caused by stored stresses in the metal when it is forged. To reduce the effect of these distortions, the camera and grid were placed at a closer distance of $L=0.38$ m from the beam and a lens with a shorter focal length (Sigma 17–50 mm f/2.8 EX DC OS HSM) was used. Since the lens had a variable focal length, the calibration to achieve an integer value of $N=9$ pixels per grid period was performed by adjusting the zoom without needing to displace the camera. Similar to the uni-axial DIC measurement, a lower frame rate of 20 kHz was used for the non-uniform beam due to the insufficient response levels (below the measurement noise floor) above 10 kHz.

The phase extraction from the images was performed via a 2D spatial convolution using a kernel of size $(2N-1)$ pixels, where *N* is the number of pixels per grid period. The kernel size was therefore 13 pixels for the uniform beam measurement and 17 pixels for the non-uniform beam. The slope fields obtained from the deflectometry technique are integrated to obtain the out-of-plane displacement for comparison with the other techniques. Both the convolution and spatial integration operations naturally smooth the data spatially. However, no explicit smoothing of the data was performed.

## Results

This section is devoted to the results obtained for the two mechanical structures. As detailed in Table 1, the chosen measurand is the transverse displacement of the beam. This requires converting acceleration, velocity and bending slopes into displacements for several of the measurement techniques. For both cases, uniform beam and non-uniform beam, the results are organized as follows. The displacement spectrum and displacement/force FRF of the two systems are compared for each of the measurement methods in order to analyze the maximum achievable frequency in the spectrum. Operational deflection shapes are extracted at resonance frequencies in order to evaluate the reconstruction of modal shapes. Frequency spectra and histograms of the measurement noise without any external excitation of the structure are also given in order to characterize the residual noise associated to each measurement technique. Finally, temporal comparisons of the transient displacement fields and vibration signals after the impact are shown in order to compare the reconstruction of time responses of the techniques.

### Uniform beam

The measurements with the uniform beam are performed with adapted excitation levels for each method. The peak impact force is 35 N for DIC and deflectometry using the manual impact hammer. For holography and LDV, peak force is 5 N with the automatic impact hammer.

#### Comparison of measured responses in the frequency domain

**Figure 8:**
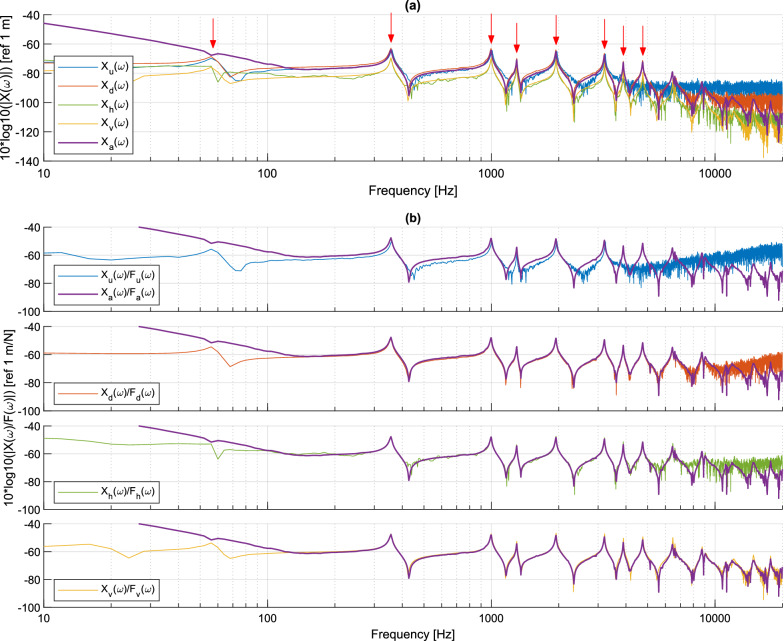
Frequency-domain response of the uniform beam near the excitation point: (**a**) displacement spectrum, with the 8 modes displayed in Fig. 10 indicated by red arrows; (**b**) displacement/force FRF comparing each technique to the accelerometer.

The frequency spectra of measured displacements are displayed in Fig. 8a and the displacement/force FRF are provided in Fig. 8b. Displacements are compared at the excitation point, which corresponds to the measurement point closest to the accelerometer for all the measurement methods. The lower displacement levels obtained with holography and vibrometry measurements are due to the lower impact force used in these two cases (see Fig. 6). The four non-contact measurement techniques are able to yield the modal response of the beam up to around 4 kHz. At higher frequency, measurements are dominated by noise, notably in DIC data and to a lesser extent, in deflectometry and holography data.

The displacements calculated with the accelerometer are very close to those obtained with the other methods, except in the low frequency range between 10 and 100 Hz. Indeed, as indicated in Table 1, the displacement spectrum is obtained by dividing the acceleration spectrum by $-\omega ^2$, which induces a difficulty at very low frequencies. DIC measurements generally exhibit a higher measurement noise, and include a slight shift in the frequencies of the anti-resonances. The largest resonance frequency observed with this method is around 3234 Hz. The measurements by deflectometry and holography are very similar, with an identification of resonances and anti-resonances in agreement with the accelerometer up to 5500 Hz. Resonant peaks are identifiable up to approximately 10 kHz. Measurements by the scanning vibrometer do agree with accelerometer results up to 10 kHz but exhibit significant noise above this frequency.

#### Analysis of the measurement noise

**Figure 9:**
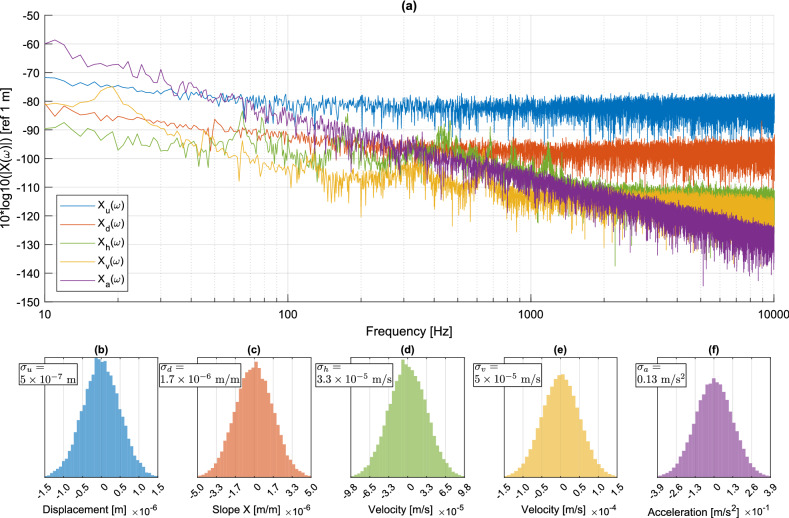
(**a**) Displacement noise spectra; (**b**–**f**) probability densities in the measurand of each technique (refer to Fig. 7 for the color code).

The level of noise in the data is one of the factors limiting the maximum frequency measurable with each measurement technique. This section investigates the frequency spectrum of the measurement noise and its probability density functions for each measurement method, at a given point at the structure surface. Note that spatial noise, i.e. the distribution of measurement noise over grid points at a given time, is not investigated in this section. The measurement noise of transverse displacements was obtained by recording data without any external force applied to the structure over a duration of 1 s. The measurement noise thus combines possible residual vibrations or drifts of the mechanical beam, photon noise, electronic noise of the image sensor (which is identical for all techniques), and the errors related to the post-processing operations of each technique. The frequency spectrum of the measurement noises at the same location as data provided in Fig. 8 are given in Fig. 9a. The spectra are provided for the displacement extracted from the raw data for each method. Data processing to reconstruct the transverse displacements follows indications in Table 1. The probability density functions of the residual noise are estimated from the original measurands, before conversion to displacements. Figure 9b–f show the probability density functions and demonstrate that, for all cases, they can be approximated by Gaussian statistics.

In agreement with the results in Figs. 8, 9a shows that, over the whole frequency range, DIC measurements exhibits a higher background noise. In addition, deflectometry measurements exhibits higher noise than holography and vibrometry, whereas the last two have a similar noise level. These noise levels are generally higher than the background noise level of the accelerometer. More quantitatively, noise in the displacement signal calculated from the accelerometer decreases with frequency, according to a slope on the order of − 20 dB/decade. This observation is consistent with the fact that the noise level of the acceleration signal is independent of the frequency. For the noise in displacement signals obtained from holographic and vibrometry measurements, the observed decay is in the order of − 10 dB/decade. The displacement noise spectrum, obtained from deflectometry and DIC measurements, are approximately flat at high frequencies, which is consistent with their respective measurands. These noise floors set the minimum values that can be measured. For example, at 1000 Hz, Fig. 9a shows that the order of magnitude of the noise floors are 100 nm for DIC, 10 nm for deflectometry and 1 nm for holography and vibrometry. From data in Fig. 9b–f, the standard deviations are estimated and we get $\sigma _u \approx 5 \times 10^{-7}$ m for DIC, $\sigma _d \approx 1.7 \times 10^{-6}$ m/m for deflectometry, $\sigma _h \approx 3.3 \times 10^{-5}$ m/s for holography, $\sigma _v \approx 5 \times 10^{-5}$ m/s for vibrometry, and $\sigma _a \approx 0.13$ m/s$^2$ for the accelerometer.

#### Operational deflection shapes

**Figure 10:**
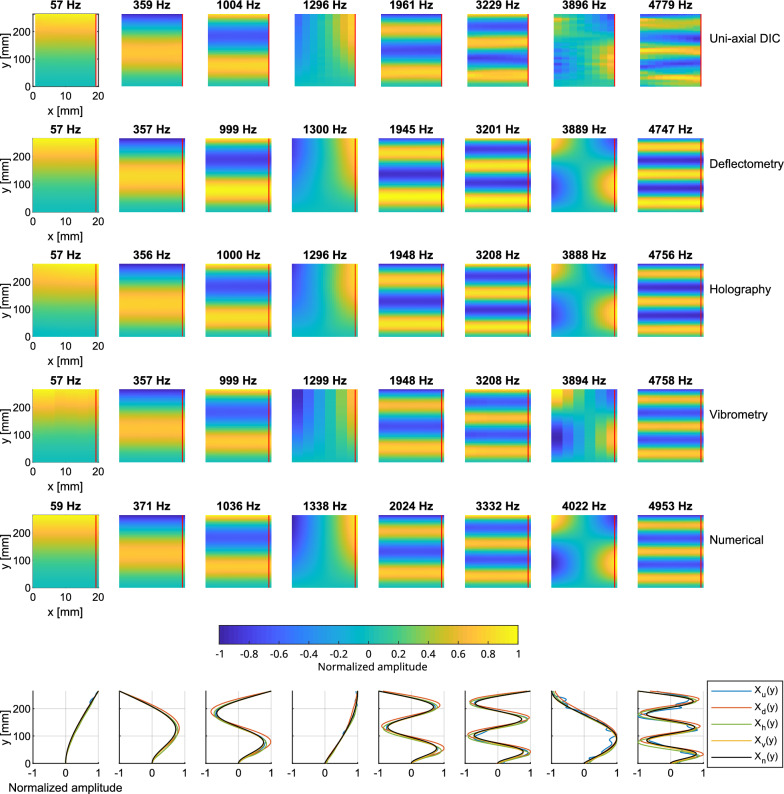
On-resonance operational deflection shapes (real part) measured on the uniform beam for the first 8 resonance frequencies indicated by arrows in Fig. 8b. The normalized amplitudes along a vertical cross-section indicated by a red line are compared for each mode shape. Deflection shapes obtained via a numerical model ($X_n$) are included for comparison.

Figure 10 shows a set of operational displacement shapes corresponding to the resonance peaks indicated by the red arrows in Fig. 8b. In addition, the mode shapes were calculated numerically using a finite element model in COMSOL and they are in good agreement with the reference vibrometry measurement. Differences in the resonance frequencies between the numerical and experimental results are due to inaccuracies in the chosen material parameters and imperfections in the clamping condition of the beam. Note that the resonance frequencies also slightly vary between measurements, and these displacement shapes correspond to the resonance peaks identified for each measurement technique. A profile along the beam axis, indicated by a vertical red line is also shown at the bottom of Fig. 10. The displacement maps are normalized by the absolute value of the largest deflection, in each case. As a consequence, the amplitude of each profile varies between $-1$ and $+1$ and takes a quasi-zero value at the clamped extremity of the mechanical beam.

The operational deflection shapes corresponding to the vibration modes of the clamped structure are observed. Due to the off-center impact, the first two torsional modes are visible at approximately 1300 Hz and 3900 Hz). The other modes are flexural modes of the mechanical beam. The resonance frequencies are found to be approximately the same for all techniques, apart for DIC measurements, which exhibit a few deviations, especially at frequencies 3324 Hz, 3900 Hz and 4788 Hz. This can be explained by an unwanted slight change in the mechanical setup. Another explanation is the fact that the area around the impact point had to be spatially interpolated because the presence of the hammer disrupted the image correlation operation. The peaks are thus selected with the FRF interpolated from surrounding points.

The deflection shapes agree quite well, especially at lower frequencies ($< 3000$ Hz) where the amplitude is higher. Deviations are observed at higher frequencies, with DIC for example, due to a low signal-to-noise ratio. This is in agreement with Fig. 8. Operational deflection shapes provided by deflectometry and holography are remarkably close.

The resolution of each technique is defined as the number of data points per length unit and is supposed to be the same in the (*x*, *y*) directions. DIC measurements use quasi-circular pixels area sized $7.9\times 6$ mm$^2$ (due to the angle of the camera) on the surface with a 2$^{nd}$ order 2D polynomial to interpolate the vibration. Measurements are here reconstructed with a resolution of $255\,\times \,12$ measurement points. This resolution leads to a density of 9.9 DPCM (Dots Per Centimeter) or 90 DPI (Dots Per Inch). The deflectometry analysis produced a measurement point for each image pixel, leading to a mesh of $925\,\times \,71$, or 35.3 DPCM ($\sim$90 DPI). However, the true spatial resolution is likely less due to smoothing by the spatial convolution operation. Holographic measurements contain $450\,\times \,31$ measurement points, leading to 16.2 DPCM ($\sim$41 DPI). For the vibrometer measurements, the beam was sampled differently in the *x* and *y* directions with $185\,\times \,7$ measurement points giving an average density of 5.3 DPCM (13.1 DPI).

#### Transient response

**Figure 11:**
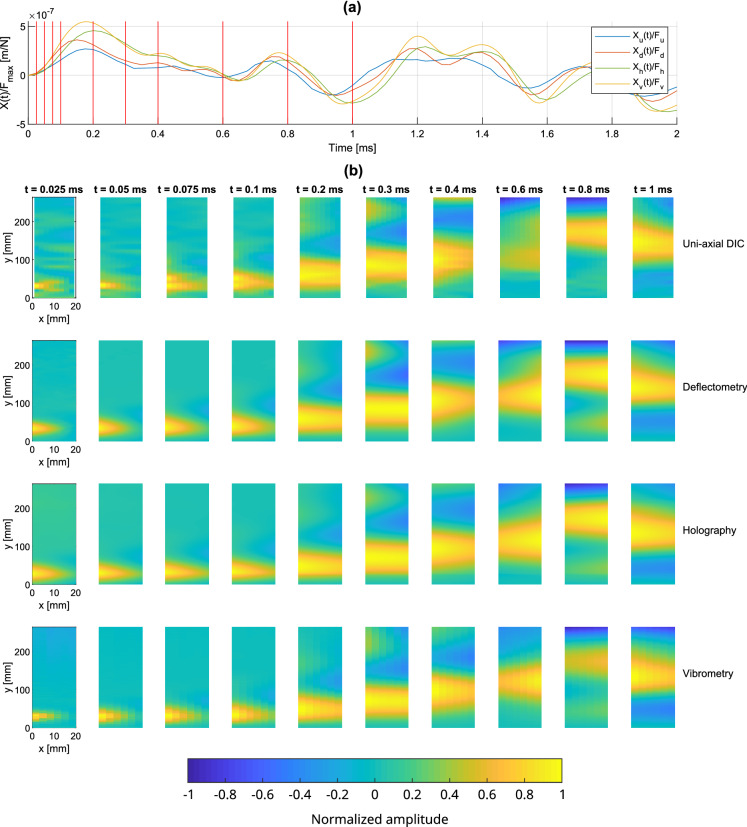
(**a**) Profiles of the temporal transient displacement responses measured with the uniform beam near the excitation point, (**b**) operational deflection shapes at 10 selected instants indicated by red lines in (**a**).

In order to appreciate the capability of the full-field techniques to accurately capture short dynamic events both in time and space, the transient response of the beam just after the impact is shown in Fig. 11. The signals are from one-shot data acquisition and no averaging is performed. The force-normalized displacement profiles captured at the accelerometer position are shown in Fig. 11a for the measurement methods. The time responses of the measurement methods are similar. DIC slightly underestimated the amplitude, consistent with Fig. 8b, and may be related to the spatially interpolated signal at the excitation point due to the presence of the hammer. Displacement maps of the beam are shown in Fig. 11b over the consecutive instants identified by the red lines in Fig. 11a. These maps are normalized at each instant for each measurement method in order to better compare them.

Similar to results presented in Fig. 10, the measured displacement maps are quite similar between the measurement methods. All the methods correctly capture the initial impact and resulting bending and torsional wave propagation. The good agreement between deflectometry and holography in the early transient response is notable. Note that interpolation artifacts/errors are visible for DIC in the area of the accelerometer on the four first maps.

### Non-uniform beam

The experiments reported in the previous section have been repeated for a similar clamped-free beam in which an Acoustic Black Hole (ABH) termination was machined (Fig. 5a). This non-uniform mechanical beam is characterized by a large contrast in vibration amplitude and wavelength between the uniform region and the ABH termination. These conditions are intended to provide a challenge for the measurement methods.

#### Comparison of the measured responses in the frequency domain

**Figure 12:**
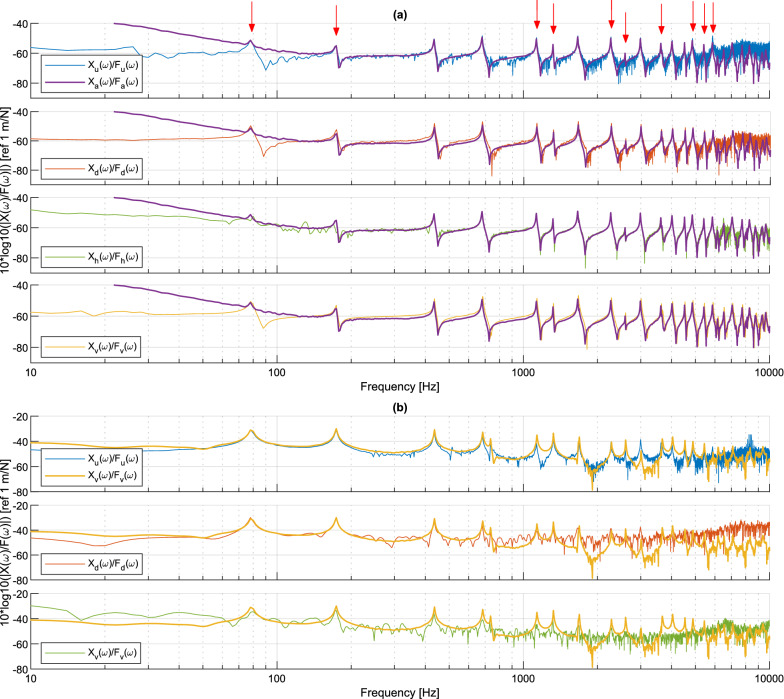
Displacement/force FRF at two positions on the non-uniform beam: (**a**) near the excitation point comparing each technique to the accelerometer, with the 10 modes displayed in Fig. 13 indicated by red arrows; (**b**) at the end of the ABH termination comparing each technique to the vibrometer.

The displacement/force FRF are shown in Fig. 12 at two positions on the beam. The first point is located at the accelerometer/excitation point near the base of the beam (Fig. 12a), while the second point is located at the end of the ABH termination (Fig. 12b). The parameters for the measurement setup are the same as for the uniform beam.

The comparison between Figs. 8b and 12a shows that the non-uniform beam has a larger modal density and a larger displacement-to-force ratio (or compliance) at the impact position than the uniform beam. The three full-field methods are able to capture resonance peaks over a larger frequency range (up to about 6000 Hz). Compared to Fig. 8b, the results provided by DIC are noisier between resonances on Fig. 12a. While a custom ink-stamp was used for making the random DIC patterns on the uniform beam, the pattern on the non-uniform beam was created with spray-paint and a stencil. The stencil did not adhere uniformly and produced a lower contrast and thus lower intensity gradients in the accelerometer region. Again, the signal had to be spatially interpolated due to the presence of the hammer. These parameters lead to a noisier measured response at the accelerometer location on the non-uniform beam.

Results in Fig. 12b at the beam end are quite different. As a general rule, the compliance increases as the thickness of the beam decreases, which translates into much larger transverse displacements in the termination of the non-uniform beam. Moreover, the displacement field varies rapidly in space. While the accuracy of DIC measurements is essentially similar to Fig. 12a, deflectometry and holography measurements are degraded. For these two methods, it is difficult to accurately capture the deformations at the end of the beam. In the case of deflectometry, the warping of the beam at the thin termination distorts the grid image observed by the camera. These distortions modify the number of pixels per grid period (a tuning factor for phase extraction; supposed constant over the entire image), producing phase errors. The local curvature of the thin end also creates a curved mirror that modifies the focus distance of the optical setup, leading to a local blurring effect that decreases precision. These phenomena are intensified by the high vibration amplitudes. For holographic measurements, it is the large difference in bending slopes between two instants that produce a high number of phase jumps in the calculated wrapped phase, making the processing and unwrapping of the phase very difficult. In order to overcome this problem, the solution would be to increase the camera’s frame rate up to 100–120 kHz so as to better sample the phase jumps from high vibration amplitudes. However, that would drastically increase the amount of data to be processed, and thus the overall computation time.

#### Operational deflection shapes

**Figure 13:**
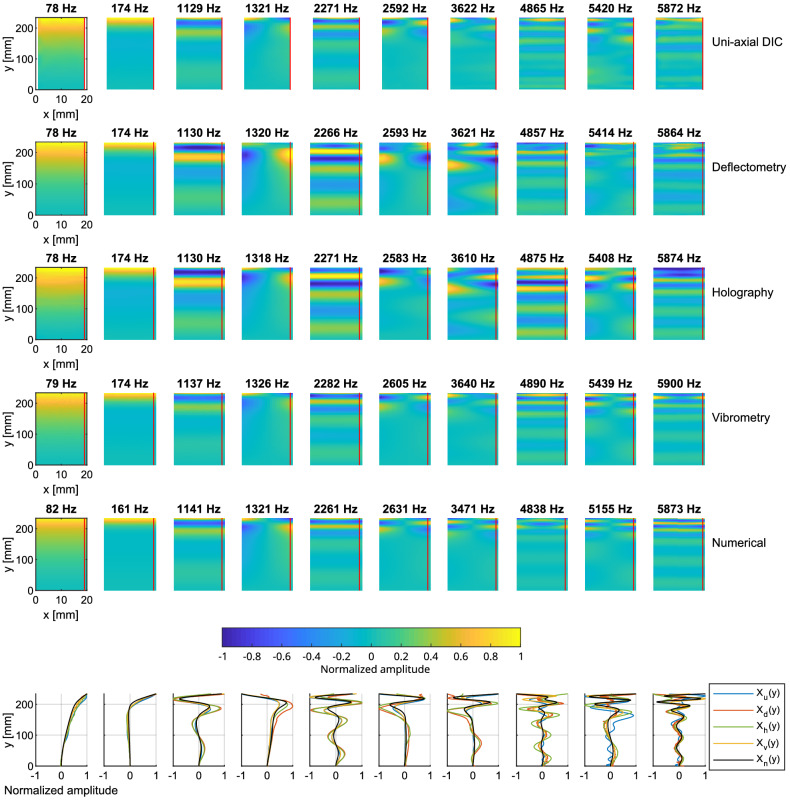
On-resonance operational deflection shapes (real part) measured on the non-uniform beam at 10 selected frequencies indicated by arrows in Fig. 12a. The normalized amplitudes along a vertical cross-section indicated by a red line are compared for each mode shape. Deflection shapes obtained via a numerical model ($X_n$) are included for comparison.

Figure 13 shows the operational displacement maps of the structure extracted at the resonance frequencies indicated by the red arrows in Fig. 12a. Again, the mode shapes were calculated numerically using a finite element model in COMSOL. It should be noted that there is no analytical solution for the flexural dynamics of the non-uniform beam. Differences are observed in the resonance frequencies between the numerical and experimental results due to inaccuracies in the chosen material parameters, uncertainties in the geometrical profile of the non-uniform beam, and imperfections in the clamping condition. Nevertheless, the numerical mode shapes show a good agreement with the reference vibrometry measurement. Profiles along the red line are also shown at the bottom of Fig. 13. DIC measurements use quasi-circular pixels sized $7\times 6$ mm$^2$ on the surface with a 2nd order 2D polynomial to interpolate the vibration field with a resolution of $238\,\times \,19$ measurement points. This resolution leads to a density of about 9.3 DPCM or 23.6 DPI. Deflectometry measurements are obtained with $925\,\times \,71$ measurement points or 35.3 DPCM (89.7 DPI), holographic measurements have $913\,\times \,61$ measurement points or 32.5 DPCM (82.6 DPI), and vibrometer measurements contain $179\,\times \,21$ measurement points or an average density of 8.6 DPCM (21.5 DPI).

The shorter wavelengths are visible in the thin part of the beam. The measured displacement maps are similar for all the measurement methods in the uniform part of the beam, but the larger discrepancies are observable in the non-uniform part. This can be explained by the fact that the ABH beam is very sensitive to environmental conditions. Thus, the experimental conditions were not exactly the same during the four experiments. DIC provides reliable results over the entire length of the beam, while deflectometry and holography have difficulties in the reconstruction for the reasons discussed above. Note the overall good agreement between DIC and vibrometry for most resonances.

#### Transient response

**Figure 14:**
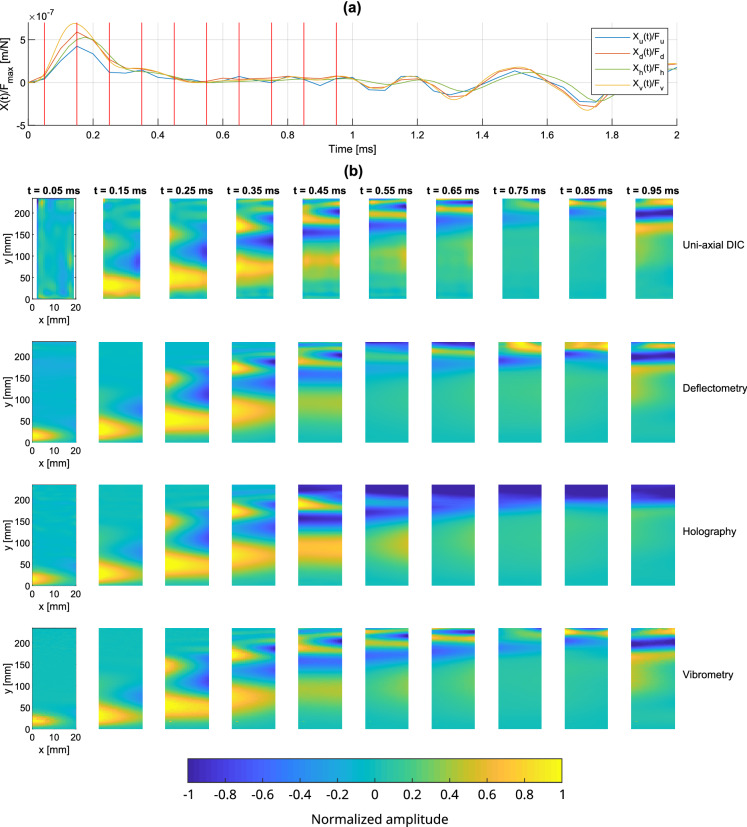
(**a**) Profiles of the temporal transient displacement responses measured on the non-uniform beam near the excitation point; (**b**) operational deflection shapes at 10 selected instants indicated by red lines in (**a**).

The transient displacement response measurements of the non-uniform beam after the impact are shown in Fig. 14. The force-normalized time profiles captured at the accelerometer position are shown in Fig. 14a. Similar to Fig. 11, the DIC measurements slightly underestimate the amplitude, likely due to the spatial interpolation of the signal as discussed previously. The normalized displacement maps are presented in Fig. 14b at the time points marked by red lines in Fig. 14a. These instants are chosen in order to observe the vibratory behavior in the different parts (uniform and non-uniform) to highlight the challenges for the methods.

The measurements at the first few instants (0.15 ms to 0.35 ms) exhibit similar results between all the measurement methods. Indeed, the lower part of the beam is uniform and does not present any particular difficulty for the methods. When the wave-front reaches the non-uniform termination at 0.45 ms, the holographic measurement fails to provide a correct estimate of the displacement and gives an inaccurate estimate of the displacement due its high amplitude, generating non spatially-resolved phase jumps. The large number of phase jumps makes impossible any de-noising and unwrapping of the Doppler phase. Deflectometry also has difficulties when the wave-front reaches the top end of the beam (at 0.55 ms) and gives incorrect estimations of the profile near the end of the beam due to the large deformations distorting the reflected grid image. On the other hand, an overall good agreement for most maps can be observed between DIC and vibrometry. These results demonstrate that DIC and laser vibrometry are robust when dealing with high vibration amplitudes such as those at the thin beam termination.

## Discussion

Table 3 shows a qualitative comparison of the different intrinsic and evaluated characteristics of the three optical measurement techniques. The purpose of this table is to provide a comprehensive and intuitive summary of the measurement abilities of each method. When questioning the choice of the adapted measurement method, the readers may refer to the table in order to use the method most suited to their problem.

Table 3 first summarizes the different characteristics of the measurements, namely the measured quantity, the temporal resolution, the maximum acquisition time and the minimum measurable quantity. Interpretations of the noise floor and the measurement dynamics that can be obtained are given. Comments related to the spatial resolution are provided, as well as the realistic dimensions of a sample that can be studied using each approach. The surface condition required for the measurements is highlighted, in addition to the setup installation and acquisition times. The last point deals with the processing time to obtain the measurand from the captured images.

**Table 3:** Qualitative comparison of the three measurement methods: digital holography, uni-axial DIC and deflectometry.

Parameter	Method		
	Holography	Uni-axial DIC	Deflectometry
Measured quantity	Displacement (out-of-plane) between two consecutive instants, usually converted to velocity.	Displacement (out-of-plane).	Bending slope (out-of-plane).
Time resolution	$1/f_{Cam}$, where $f_{Cam}$ is the frame rate of the camera.		
Max acquisition time	Depends on image size and on-board camera memory; typically a few seconds of 1 Mpix images at 10 kHz. Low-speed ($\sim$1 kHz) applications can use off-board storage for much longer acquisitions.		
Minimum measurable quantity	$\approx 2 \pi /160$ for the Doppler phase between two consecutive instants; for conversion to velocity refer to Eq. (4); about 1 nm in this study (after conversion to displacement).	$\approx 5\times 10^{-4} L_{s} / (N_{p} sin(\theta ))$, where $\theta$ is the angle between optical and displacement axes, $L_{s}$ the size of the measured surface, $N_{p}$ the number of available pixels; about 100 nm in this study.	$\approx p/4\pi L$, where *p* is the grid pitch and *L* is the grid-sample distance; about 10 nm in this study (after conversion to displacement).
Parameters influencing the noise floor	Speckle decorrelation; depends on local phase jump density and orientation, and sensor dimensions.	Quality and contrast of projected pattern, number of pixels, size of the measured surface, photon noise.	Quality of reflection and the number of pixel per grid period in the recorded image.
Dynamic range	From few nm to about 10 μm (depending on spatial sampling of phase jumps; at least 4 pixels per phase jump in the Doppler phase are required).	From few hundreds of nm to few cm, as long as the surface is imaged by the cameras and the angle (optical axis/normal of the surface) is not too large.	From few tens of nm to a few mm, provided the bending curvature is not too large.
Spatial resolution	Depends on the structure-to-sensor distance, pixel pitch, number of pixels of the sensor and wavelength of light; about 0.3 mm in this study.	Depends on the pattern and sensor resolution; optimal configuration, $\approx$ 1 point every 1–3 pixels; about 1 mm in this study.	Depends on the number of pixels per grid period in the image; about 0.3 mm in this study.
Sample dimensions	From mm$^2$ to 500 cm$^2$.	From few cm$^2$ to several m$^2$, with adapted calibration procedure.	From cm$^2$ up to about 1 m$^2$, provided the observed grid pattern is large enough.
Sample surface preparation	Non-depolarizing diffuse reflection.	Speckle/random pattern.	Specular reflection (defined by Rayleigh criterion).
Setup installation time	$\approx$ 4 h, negligible surface preparation.	$\approx$ 1–2 h with surface preparation.	$\approx$ 30 mins, not including surface preparation (depends on material).
Acquisition time	Depends on the studied phenomenon and regime (stationary or transient); typically a few seconds.		
Image download time	Depends on the camera and the storage media (HDD, SSD); typically 5 minutes for 10,000 images.		
Processing time	$\approx$ 12 h for 10,000 images.	A few mins for 10,000 images.	$\approx$ 1 h for 10,000 images.

The three full-field optical methods possess a significant advantage over scanning LDV: the data at all spatial points is acquired simultaneously, which drastically reduces the acquisition time, thus suppressing the mechanical drift/variations due to long acquisition times and enables the possibility of measuring non-repeatable phenomena. While the laser Doppler vibrometer is a long-standing method, with hardware and software that have been optimized for decades, the three full-field optical methods are relatively new for vibration measurement applications. It follows that they need to be improved in the near future, especially in terms of user-friendliness, software and computation time.

First, one key element is the preparation of the surface. Digital holography requires a diffuse reflection that is non-depolarizing due to the coherent laser source. This can be quickly achieved with certain white or metallic spray paints. Until recently, scanning LDV also needed a diffusely reflective surface. However, since the emergence of infrared lasers, many surfaces can be measured without specific preparation. DIC requires a speckle/random pattern that is painted or projected onto the surface^79^ and is applicable to more complex 3D geometries. Deflectometry measures mirror-like surfaces, but with adapted infrared cameras, some metallic surfaces may be inspected with almost no preparation.

Secondly, considering the hardware and setup, all-in packages are available for scanning LDV (laser system, acquisition board, acquisition and processing software). Commercial systems also exist for DIC measurements and are usually intended for 2D measurements using a single camera, or 3D measurements (including out-of-plane) using two cameras. Meanwhile, the uni-axial DIC approach applied in this study enabled out-of-plane displacements to be obtained a single high-speed camera. This shows that there is still a interest for developing in-house software for specific applications such as vibration analysis. For the two other methods, an adapted setup and software has to be designed. While the deflectometry setup is relatively simple, digital holography requires an optical table, laser source, and optical elements that must be accurately arranged. In the near future, holographic imaging could be included in a packaged optical head, which would simplify and optimize the practical implementation.

Finally, to perform full-field optical measurements, one usually has to deal with the camera controller software, which are not always fully adapted to the experimental method. Accurate synchronisation of the optical measurements with other signals can be difficult. Some camera hardware can measure the time delay between the trigger signal and the first acquired frame. Otherwise, the time shift needs to be estimated by independently recording the camera exposure signal. Furthermore, image transfer rates can be time-consuming depending on the camera, software or storage device.

Note that the full-field methods considered in this paper may use two types of cameras. On the one hand, high-speed cameras enable measuring transient and non-repeatable phenomena, but they may include cooling fans that cannot be turned off during acquisition, therefore generating unwanted vibration of the optical system. With most high-speed cameras, there is also typically a trade-off between the frame rate and image size, thus diminishing the spatial resolution (or number of measurement points) at higher sampling rates. On the other hand, industrial low frame rate cameras can be used for lock-in measurements with several stationary excitation signals, even if they cannot be used for transient phenomena. Such cameras have high spatial resolutions and low noise sensors, thus improving the overall accuracy of the measurement. As a general rule, camera control libraries are available allowing users to build custom software to facilitate experiments.

## Conclusion

This paper provides a quantitative and qualitative evaluation of three full-field optical measurement techniques (digital holography, uni-axial digital image correlation (DIC), and deflectometry) in the context of vibration analysis. The comparisons presented in this paper highlighted the strengths and weaknesses of each measurement method through two case studies involving a uniform cantilever beam (basic measurement case) and a non-homogeneous cantilever beam generating a large dynamic of vibration amplitude and wavelength. The tested structures were excited by a off-center hammer impact near the clamped end of the beam. Two classical reference measurements were also implemented, namely a scanning laser Doppler vibrometer and accelerometer.

Results with the uniform beam show that the three methods are able to measure the propagation of bending waves resulting from a hammer impact on the structure. The temporal data can be used to perform modal analysis by extracting frequency spectra on the whole structure and the beam’s operational mode shapes. The three full-field approaches provide high-resolution spatial measurements at all points from a single impact excitation, with an acquisition time on the order of 1 second. In comparison, the laser vibrometer must measure a repeatable impact for each measurement point, leading to lengthy acquisitions (multiple hours) for an equivalent time and spatial resolution. Deflectometric and holographic measurements also demonstrate high accuracy and low noise level for such transient measurements.

Results with the non-uniform beam show that the three techniques are able to perform accurate measurements on the uniform part of the beam. However, only uni-axial DIC is able to study the thin termination. Deflectometry is disturbed by the non-planarity of the structure (the measured surface should be as flat as possible). Holography is disturbed by the very large amplitude of the vibration response leading to a huge number of phase jumps and a high noise level making de-noising and unwrapping operations impossible to perform. A possible solution would be to increase the camera frame rate up to 100-120 kHz, but at the cost of the huge amount of data to be processed. Uni-axial DIC takes advantage of high vibration level to provide accurate measurements because the method is intrinsically more robust to high vibration levels.

The advantages and drawbacks highlighted in this paper are listed in a comparison table, which yields the reader an intuitive summary of each measurement’s capability. The text also includes recommendations for the practical implementation of the techniques, allowing experimenters to choose the method most adapted to their application.

## Data Availability

The datasets of the measurements conducted during the current study are available from the corresponding author upon reasonable request.

## References

[1] Drain LE (1980). The Laser Doppler Technique.

[2] Scruby, C. B. & Drain, L. E. *Laser Ultrasonics: Techniques and Applications* (1990).

[3] Monchalin JP, Thompson DO, Chimenti DE (1993). Progress towards the application of laser-ultrasonics in industry. Review of Progress in Quantitative Nondestructive Evaluation: Volumes 12A and 12B.

[4] Castellini P, Revel GM, Tomasini EP (1998). Laser Doppler vibrometry : A review of advances and applications. Shock Vibr. Dig.

[5] MacPherson WN (2007). Multipoint laser vibrometer for modal analysis. Appl. Opt..

[6] Sun K (2014). Scanning laser-line source technique for nondestructive evaluation of cracks in human teeth. Appl. Opt..

[7] Connelly MJ (2008). Multipoint laser Doppler vibrometry using holographic optical elements and a CMOS digital camera. Opt. Lett..

[8] Fu Y, Guo M, Phua PB (2011). Multipoint laser Doppler vibrometry with single detector: Principles, implementations, and signal analyses. Appl. Opt..

[9] Fu Y, Guo M, Phua PB (2010). Spatially encoded multibeam laser Doppler vibrometry using a single photodetector. Opt. Lett..

[10] Johansmann, M. & Sauer, J. A new tool for three dimensional non-contact vibration measurements in automotive applications. Tech. Rep., SAE Technical Paper 10.4271/2005-26-052 (2005).

[11] Baqersad J, Poozesh P, Niezrecki C, Avitabile P (2017). Photogrammetry and optical methods in structural dynamics—A review. Mech. Syst. Signal Process..

[12] Tiwari V, Sutton MA, McNeill SR (2007). Assessment of high speed imaging systems for 2D and 3D deformation measurements: Methodology development and validation. Exp. Mech..

[13] Reu PL, Rohe DP, Jacobs LD (2017). Comparison of DIC and LDV for practical vibration and modal measurements. Mech. Syst. Signal Process..

[14] Beberniss TJ, Ehrhardt DA (2017). High-speed 3D digital image correlation vibration measurement: Recent advancements and noted limitations. Mech. Syst. Signal Process..

[15] Durand-Texte T, Simonetto E, Durand S, Melon M, Moulet MH (2019). Vibration measurement using a pseudo-stereo system, target tracking and vision methods. Mech. Syst. Signal Process..

[16] Durand-Texte T, Melon M, Simonetto E, Durand S, Moulet MH (2020). Single-camera single-axis vision method applied to measure vibrations. J. Sound Vib..

[17] Huang L, Idir M, Zuo C, Asundi A (2018). Review of phase measuring deflectometry. Opt. Lasers Eng..

[18] Surrel Y, Fournier N, Grédiac M, Paris P-A (1999). Phase-stepped deflectometry applied to shape measurement of bent plates. Exp. Mech..

[19] Giraudeau A, Pierron F, Guo B (2010). An alternative to modal analysis for material stiffness and damping identification from vibrating plates. J. Sound Vib..

[20] Xavier J (2013). Characterisation of the bending stiffness components of MDF panels from full-field slope measurements. Wood Sci. Technol..

[21] Kim JH, Pierron F, Wisnom MR, Syed-Muhamad K (2007). Identification of the local stiffness reduction of a damaged composite plate using the virtual fields method. Compos. Part A Appl. Sci. Manuf..

[22] Devivier C, Pierron F, Wisnom MR (2013). Impact damage detection in composite plates using deflectometry and the Virtual Fields Method. Compos. Part A Appl. Sci. Manuf..

[23] Giraudeau A, Pierron F (2010). Measurement of vibrating plate spatial responses using deflectometry and high speed camera. AIP Conf. Proc..

[24] O’Donoughue, P., Robin, O. & Berry, A. Measuring the vibration response of plane panels under stationary and transient mechanical excitations using deflectometry. In *INTER-NOISE NOISE-CON Congr. Conf. Proc.*, vol. 253, 4686–4692 (2016).

[25] Devivier C, Pierron F, Glynne-Jones P, Hill M (2016). Time-resolved full-field imaging of ultrasonic Lamb waves using deflectometry. Exp. Mech..

[26] O’Donoughue P, Robin O, Berry A (2018). Time-resolved identification of mechanical loadings on plates using the virtual fields method and deflectometry measurements. Strain.

[27] O’Donoughue P, Robin O, Berry A (2019). Time-space identification of mechanical impacts and distributed random excitations on plates and membranes. Proc. Inst. Mech. Eng. Part C J. Mech. Eng. Sci..

[28] Kaufmann R, Ganapathisubramani B, Pierron F (2019). Full-field surface pressure reconstruction using the virtual fields method. Exp. Mech..

[29] Toniuc H, Pierron F (2019). Infrared deflectometry for slope deformation measurements. Exp. Mech..

[30] Robin O, O’Donoughue P, Berry A, Farley V, Prithipaul K (2021). Full field vibration measurements on a cantilever beam under impact using visible and infrared deflectometry. Appl. Acoust..

[31] Kreis, T. *Holographic Interferometry: Principles and Methods*. No. 1 in Akademie Verlag series in optical metrology, 1 edn (Akad.-Verl, 1996).

[32] Picart, P. (ed.) *New Techniques in Digital Holography*. Instrumentation and measurement series (ISTE Ltd, Wiley, 2015).

[33] Hazell CR, Liem SD (1973). Vibration analysis of plates by real-time stroboscopic holography. Exp. Mech..

[34] Leval J, Picart P, Boileau JP, Pascal JC (2005). Full-field vibrometry with digital Fresnel holography. Appl. Opt..

[35] Picart P (2007). Tracking high amplitude auto-oscillations with digital Fresnel holograms. Opt. Express.

[36] Alexeenko I, Gusev M, Gurevich V (2009). Separate recording of rationally related vibration frequencies using digital stroboscopic holographic interferometry. Appl. Opt..

[37] De Greef D, Soons J, Dirckx JJ (2014). Digital stroboscopic holography setup for deformation measurement at both quasi-static and acoustic frequencies. Int. J. Optomechatronics.

[38] Pedrini G, Tiziani HJ, Zou Y (1997). Digital double pulse-TV-holography. Opt. lasers Eng..

[39] Pedrini G, Fröning PH, Fessler H, Tiziani HJ (1998). Transient vibration measurements using multi-pulse digital holography. Opt. Laser Technol..

[40] Foltete, E., Piranda, J. & Raynaud, J. L. Quantitative dynamical measurements for model updating using electronic speckle interferometry. In *Proceedings of SPIE, the International Society for Optical Engineering*, vol. 4359, 1305–1310 (Society of Photo-Optical Instrumentation Engineers, 2001).

[41] Chambard JP, Chalvidan V, Carniel X, Pascal JC (2002). Pulsed TV-holography recording for vibration analysis applications. Opt. Lasers Eng..

[42] Trillo C (2003). Measurement of the complex amplitude of transient surface acoustic waves using double-pulsed TV holography and a two-stage spatial Fourier transform method. Meas. Sci. Technol..

[43] Georges, M. *et al.* Double-pulsed holographic interferometry with photorefractive crystals for vibration and shock analysis. In *Photorefractive Effects, Materials, and Devices*, 661, 10.1364/PEMD.2005.661 (OSA, 2005).

[44] Aguayo DD (2010). Insect wing deformation measurements using high speed digital holographic interferometry. Opt. Express.

[45] Solís SM, Santoyo FM, Hernández-Montes M (2012). 3D displacement measurements of the tympanic membrane with digital holographic interferometry. Opt. Express.

[46] Samson B, Verpillat F, Gross M, Atlan M (2011). Video-rate laser Doppler vibrometry by heterodyne holography. Opt. Lett..

[47] Khaleghi M, Guignard J, Furlong C, Rosowski JJ (2015). Simultaneous full-field 3-D vibrometry of the human eardrum using spatial-bandwidth multiplexed holography. J. Biomed. Opt.

[48] Kakue T (2017). Digital holographic high-speed 3D imaging for the vibrometry of fast-occurring phenomena. Sci. Rep..

[49] Poittevin J, Picart P, Faure C, Gautier F, Pézerat C (2015). Multi-point vibrometer based on high-speed digital in-line holography. Appl. Opt..

[50] Meteyer E (2021). Lock-in vibration retrieval based on high-speed full-field coherent imaging. Sci. Rep..

[51] Rothberg SJ (2017). An international review of laser doppler vibrometry: Making light work of vibration measurement. Opt. Lasers Eng..

[52] Lutzmann P, Gohler B, Hill CA, van Putten F (2016). Laser vibration sensing at fraunhofer IOSB: Review and applications. Opt. Eng..

[53] Halkon BJ, Rothberg SJ (2017). Taking laser doppler vibrometry off the tripod: Correction of measurements affected by instrument vibration. Opt. Lasers Eng..

[54] Castellini P, Martarelli M, Tomasini EP (2006). Laser doppler vibrometry: Development of advanced solutions answering to technology’s needs. Mech. Syst. Signal Process.

[55] Fu Y, Guo M, Phua P (2011). Multipoint laser doppler vibrometry with single detector: Principles, implementations, and signal analyse. Appl. Opt..

[56] Di Maio D (2021). Continuous scanning laser vibrometry: A raison d’être and applications to vibration measurements. Mech. Syst. Signal Process..

[57] Margerit P, Gobin T, Lebée A, Caron JF (2021). The robotized laser doppler vibrometer: On the use of an industrial robot arm to perform 3d full-field velocity measurements. Opt. Lasers Eng..

[58] Gabor D (1948). A new microscopic principle. Nature.

[59] Leith EN, Upatnieks J (1961). New techniques in wavefront reconstruction. J. Opt. Soc. Am.

[60] Poon TC (2006). Digital Holography and Three-dimensional Display.

[61] Lagny L (2019). Visualization of travelling waves propagating in a plate equipped with 2D ABH using wide-field holographic vibrometry. J. Sound Vib..

[62] Goodman JW (2005). Introduction To Fourier Optics.

[63] Kemao Q, Gao W, Wang H (2008). Windowed Fourier-filtered and quality-guided phase-unwrapping algorithm. Appl. Opt..

[64] Kemao Q (2007). Two-dimensional windowed Fourier transform for fringe pattern analysis: Principles, applications and implementations. Opt. Lasers Eng..

[65] Montresor S, Tahon M, Laurent A, Picart P (2020). Computational de-noising based on deep learning for phase data in digital holographic interferometry. APL Photonics.

[66] Xia H, Guo RX, Fan ZB, Cheng HM, Yang BC (2012). Non-invasive mechanical measurement for transparent objects by digital holographic interferometry based on iterative least-squares phase unwrapping. Exp. Mech..

[67] Xia H (2016). Phase calibration unwrapping algorithm for phase data corrupted by strong decorrelation speckle noise. Opt. Express.

[68] Hild F, Roux S (2006). Digital image correlation: From displacement measurement to identification of elastic properties—A review. Strain.

[69] Sutton MA, Orteu JJ, Schreier H (2009). Image Correlation for Shape, Motion and Deformation Measurements.

[70] Yu L, Pan B (2017). Single-camera high-speed stereo-digital image correlation for full-field vibration measurement. Mech. Syst. Signal Process..

[71] Durand-Texte T, Melon M, Simonetto E, Durand S, Moulet MH (2018). 3d vision method applied to measure the vibrations of non-flat items with a two-mirror adapter. J. Phys. Conf. Ser..

[72] Durand-Texte T, Simonetto E, Durand S, Melon M, Moulet MH (2017). Estimation of the uncertainties of a method of measuring vibration deformations by 3D vision. Instrumentation Mesure Métrologie.

[73] Devivier C, Pierron F, Wisnom MR (2012). Damage detection in composite materials using deflectometry, a full-field slope measurement technique. Compos. Part A Appl. Sci. Manuf..

[74] Shepherd MR, Robin O, Hambric S, O’Donoughue P (2018). Estimating Poisson’s ratio of a free, rectangular panel using video-based modal analysis. J. Acoust. Soc. Am..

[75] Grédiac M, Sur F, Blaysat B (2016). The grid method for in-plane displacement and strain measurement: A review and analysis: the grid method. Strain.

[76] Pelat A, Gautier F, Conlon SC, Semperlotti F (2020). The acoustic black hole: A review of theory and applications. J. Sound Vib..

[77] Denis V, Pelat A, Gautier F, Elie B (2014). Modal Overlap Factor of a beam with an acoustic black hole termination. J. Sound Vib..

[78] Picart P, Li JC (2012). Digital Holography.

[79] Etchepareborda P, Moulet MH, Melon M (2021). Random laser speckle pattern projection for non-contact vibration measurements using a single high-speed camera. Mech. Syst. Signal Process..

